# Matched sampling reveals uncoupled phenotypic plasticity during metastasis formation in uveal melanoma

**DOI:** 10.1016/j.isci.2026.116397

**Published:** 2026-06-19

**Authors:** Karim Al-Ghazzawi, Sabrina Borchert, Siyang Liu, Amal R. Al Kadi, Fung-Yi Cheung, Georgia Antonopoulou, Tobias Blau, Andreas Junker, Leyla Jabbarli, Sylvia Hartmann, Olaf D. Muras, Michael Zeschnigk, Tobias Kiefer, Kathy Keyvani, Michael Wessolly, Miltiadis Fiorentzis, Utta Berchner-Pfannschmidt, Nikolaos Bechrakis, Fabian Mairinger, Halime Kalkavan

**Affiliations:** 1 Department of Ophthalmology, University Hospital Essen, University of Duisburg-Essen, Essen, Germany; 2 Department of Pathology, University Hospital Essen, University of Duisburg-Essen, Essen, Germany; 3 Institute of Neuropathology, University Medicine Essen, University of Duisburg-Essen, Essen, Germany; 4 Institute of Human Genetics, University Hospital Essen, University of Duisburg-Essen, Essen, Germany; 5 Medical Faculty, University Duisburg-Essen, 45122 Essen, Germany; 6 West German Cancer Center, Department of Medical Oncology, University Hospital Essen, Essen, Germany; 7 German Cancer Consortium (DKTK), Partner Site University Hospital Essen, Essen, Germany; 8 National Center for Tumor Diseases (NCT) West, Campus Essen, 45122 Essen, Germany; 9 Spatiotemporal Tumor Heterogeneity, German Cancer Consortium (DKTK), Partner Site Essen, A Partnership Between German Cancer Research Center (DKFZ) and University Hospital Essen, Essen, Germany; 10 Division of Solid Tumor Translational Oncology, DKTK, Essen, Germany; 11 Bridge Institute of Experimental Tumor Therapy, West German Cancer Center, University Hospital Essen, University of Duisburg-Essen, Essen, Germany

**Keywords:** Immune system, Cancer systems biology, Cancer

## Abstract

Uveal melanoma (UM) is a genetically well-defined yet phenotypically diverse malignancy with a strong predilection for liver metastasis. To define phenotypic divergence during progression, we performed comprehensive gene expression profiling of primary UM (pUM) and metastatic UM (mUM) samples. Two principal molecular subtypes emerged in both entities. Matched pUM-mUM samples showed little transcriptional concordance, indicating extensive reprogramming during metastasis. Approximately half of the pUM samples exhibited a proliferative, MAPK/PI3K-driven phenotype, suggesting heterogeneous dependency on PKC signaling. Immune landscapes also diverged; pUM was dominated by adaptive responses, whereas mUM displayed innate signaling and macrophage-driven immunosuppression. The inflammatory pUM-A subtype was associated with poor survival, consistent with The Cancer Genome Atlas (TCGA) data linking chromosomal aberrations such as monosomy 3 to progression. Analysis of 53 UM samples demonstrated context-dependent phenotypes, supporting site-specific, phenotype-guided therapeutic strategies integrating pathway inhibition and immune modulation.

## Highlights

• RNA-seq identifies two distinct subtypes in each primary and metastatic UM
• Matched sampling reveals phenotypic reprogramming during UM metastasis
• Immune landscapes shift from adaptive in primary UMs to innate in metastatic UMs
• The inflammatory primary UM subtype correlates with worse patient survival

## Graphical abstract

**Figure:**
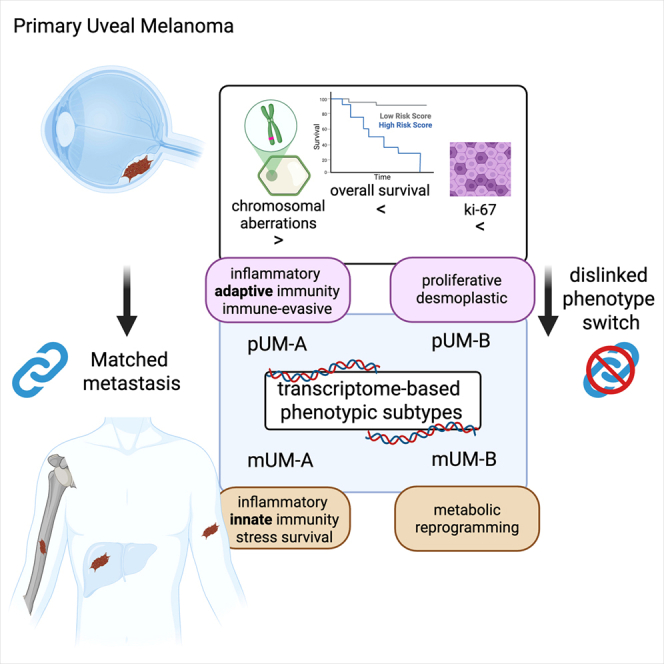

## Introduction

Primary uveal melanoma (pUM) is the predominant primary intraocular malignancy among adults. It originates in the uvea, a layer of tissue that includes the iris, ciliary body, and choroid. Despite being the most common intraocular tumor manifestation in adults, the disease itself is quite rare, with an annual incidence of around 6 cases per million population in Europe and the USA.^1^ Enucleation has largely been replaced by diverse forms of eye preservation, namely radiotherapy, phototherapy, and local tumor resection, which, sometimes, are even administered in combination. Optimal ocular outcomes are associated with small tumors that do not extend near the optic disc and/or fovea.^2^ While the primary objective of local, ocular treatment is the preservation of the eye and maintaining vision, increasing efforts have been aimed to implement (neo-)adjuvant treatment strategies to prevent metastatic disease.

Almost every second patient with pUM develops metastatic disease. In more than 90% of these patients with metastatic UM (mUM), the liver is affected even years after treatment of the primary tumor in the eye.^3^ Tumors with a height as small as 2 mm can metastasize,^4^ underscoring the need for a better understanding of the pathophysiology of metastasis in uveal melanomas (UMs). Overall, UM metastases exhibit less responsiveness to chemotherapy or immune checkpoint inhibitors compared with cutaneous melanoma, most likely due to their low mutational burden.^5^ Some therapeutic approaches have emerged as, for instance, partial hepatectomy for solitary metastases, percutaneous or intraarterial hepatic perfusion with melphalan, and targeted immunotherapies.^6^ Yet, median overall survival (OS) of patients with mUM is still very limited, even with effective treatments like the gp100-derived peptide-targeting bispecific antibody tebentafusp, with median OS of approximately 15 months compared with 10 months in the control group.^7^

Consequently, while a number of reliable biomarkers have been discovered to identify patients at high risk of developing metastatic disease, there remains a critical need for the characterization of phenotypic profiles that help guide neoadjuvant and adjuvant therapeutic strategies. Genomic studies based on expression data have reported that UM could be first subdivided into two main groups^8^ and, recently, into four main groups using unsupervised hierarchical clustering according to genetic alterations (comprehensive multiplatform analysis), pointing out a poor prognosis subtype.^9^ Yet, to date, the molecular signatures and expression patterns associated with UM remain not fully understood. For instance, in contrast to other malignancies, expression of the human leukocyte antigen (HLA) class I shows an inverse correlation with patient survival in UM patients, where low HLA class I expression is associated with better survival.^1^ Increased expression levels of HLA class I are associated with a pro-tumoral, inflammatory phenotype involving an infiltration by M2 type macrophages and lymphocytes.^10^^,^^11^^,^^12^ It has been suggested that genetic changes, specifically the loss of BAP1 expression, is associated with further increase in the influx of such immune cells, creating a pro-tumoral immune-microenvironment.^13^

Utilizing matched samples from primary tumors and liver metastases presents a distinct and precise research method, allowing for an in-depth examination of the relationship between gene expression signatures and the development of metastasis. Despite its potential, this approach has not been investigated further in previous mUM studies, highlighting the novelty of the current research. With better understanding of molecular patterns of UM metastases compared to their primary UM counterparts, we intend to test the hypotheses that (1) pUMs and mUMs exhibit distinct transcriptomic phenotypes despite shared genetic drivers, and (2) primary tumor phenotypes do not predict metastatic phenotypes, reflecting significant phenotypic plasticity during metastasis.

## Results

### Transcriptomic profiling of pUM and mUM samples reveals distinct molecular phenotypes

**Figure 1:**
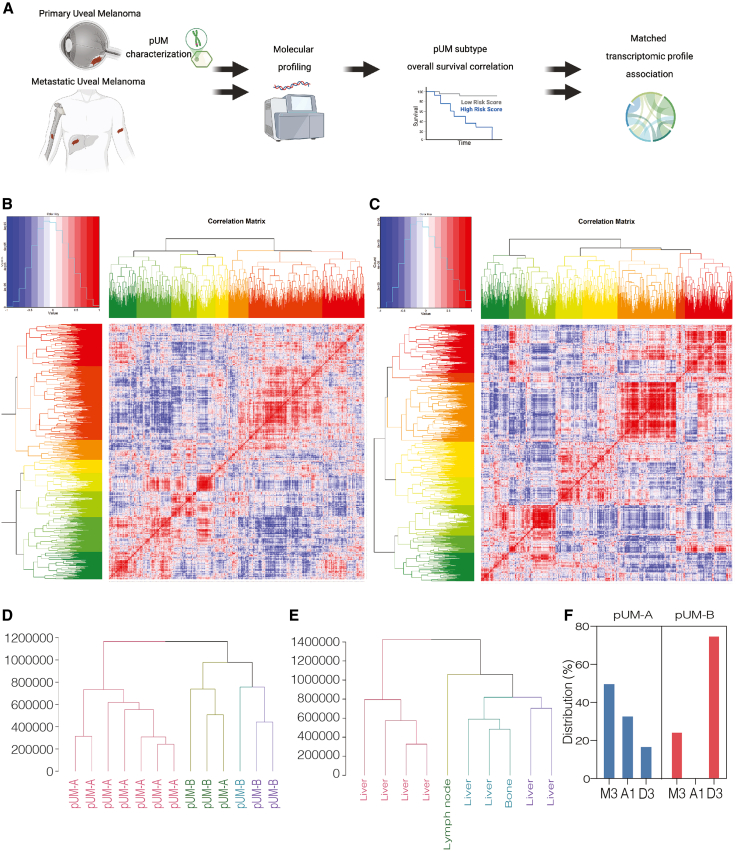
Determination of molecular biological phenotype of the primary tumor and metastatic tumor (A) Experimental design showing methodology with the analysis of genetic aberrations, transcriptomic profiling, survival, and matched transcriptomic profile association of primary uveal melanoma (pUM) and metastatic uveal melanoma (mUM). (B and C) Heatmaps showing clusters of biological phenotypes of the primary tumor (B) or metastatic tumor (C) and corresponding gene expression (side bars are individual genes, and matrix depicts genes’ fold-change (FC) upregulation [red] or downregulation [blue]). (D and E) Unsupervised hierarchical clustering of transcriptomic data with resulting gene signatures of (D) pUM samples (pUM-A, *n* = 8; pUM-B, *n* = 5) and (E) organ metastases (*n* = 8 liver, *n* = 1 bone, *n* = 1 lymph node). The *y* axis depicts the height of the branches indicating dissimilarity between the clusters. (F) Bar chart illustrating the mutational status of pUM determined by microsatellite analysis (MSA) of chromosome 3, 6, and 8 aberrations in the pUM samples. pUM-A samples: monosomy 3 (M3), 50%; disomy 3 (D3), 17.7%; allelic imbalance (AI), 33.3%; pUM-B samples: M3, 25%; D3, 75%; AI, 0%.

In total, 23 tumor samples were analyzed for transcriptomic profiling. Primary tumors included 13 enucleated UMs without any prior therapy. Ten samples were derived from metastatic sites from patients with matched evaluable primary tumors. A detailed overview of the discovery and matched cohort analyses is provided in Figure S1. First, we conducted independent unsupervised hierarchical clustering on the transcriptome data generated with HTG EdgeSeq technology. This analysis identified distinct molecular phenotypes for both the primary tumors (pUM-A and pUM-B) and metastatic tumors (mUM-A and mUM-B) (Figures 1A–1E). Although most metastatic samples were derived from the liver (8 of 10), unsupervised hierarchical clustering separated their genetic profiles in a balanced manner, with *n* = 4 in each of the two groups (Figure 1E). The transcriptome of one bone metastasis showed similar gene signature to liver metastases within the same group, while a lymph node metastasis separated early from the remaining cohort.

Next, we sought to gain further insights into the clinical and biological differences between the two pUM molecular subtypes identified by unsupervised hierarchical clustering (Table 1). Two pUM samples that had initially been assigned to pUM subtypes by unsupervised clustering were excluded from further analysis after they failed statistical validation. Immunohistochemistry of primary tumors revealed a slightly higher mean Ki-67 expression in pUM-B (32%) than in pUM-A (19%). Yet, the mean tumor volume in pUM-A was higher, at 1,700 mm^3^, than only 1240 mm^3^ in pUM-B samples. Chromosomal aberrations are a well-established prognosticator in UMs.^14^ In particular, allelic imbalances (AIs) including monosomy 3 (M3) are correlated with worse overall patient survival. Hence, we performed microsatellite analysis of all primary tumors. Notable differences were observed, with pUM-B showing predominantly disomy 3 (D3) status (75%), while tumors of pUM-A subtype were frequently correlated with chromosomal aberrations including M3 (50%) and AIs (33%) (Figure 1F).

**Table 1:** Clinical characteristics of patients stratified by molecular subtype

Characteristics	pUM-A	pUM-B	*p*	Statistical test
Patients	*n* = 6 (46%)	*n* = 5 (38%)	–	–

**Sex**				

Female	66%	60%	0.87	chi-squared test^2^: 0.025
Male	34%	40%	v	–

**Age at diagnosis (years)**				

Mean (range)	64 (44–76)	56 (43–70)	0.13	Mann-Whitney U test
Tumor basal diameter (mm), mean ± SD	14.8 ± 4.8	11.6 ± 4.5	–	–
Tumor height (mm), mean ± SD	10.3 ± 3.7	9.8 ± 4.5	–	–
Tumor volume (mm^3^), mean ± SD	1700 ± 940	1244 ± 790	0.41	one-sample *t* test

**Ciliary body involvement, (%)**				

absent	33.3%	40%	0.83	chi-squared test^2^: 4.3
present	66.7%	60%	–	–
Mitosis (KI-67), mean ± SD	19% ± 13%	32% ± 16%	0.41	one-sample *t* test

**Chromosomal abnormalities, (%)**				

M3	50%	25%	–	–
AI	33%	0%	0.27	chi-squared test^2^: 0.06
D3	17%	75%	–	–

Taken together, clinical parameters of primary tumors suggest a more aggressive subtype in pUM-A than in pUM-B due to overall larger tumors and a higher incidence of chromosomal aberrations. However, larger sample sizes are required to confirm the statistical significance of our observations.

### Chromosomal aberrations correlate with pUMs of inflammatory phenotype

**Figure 2:**
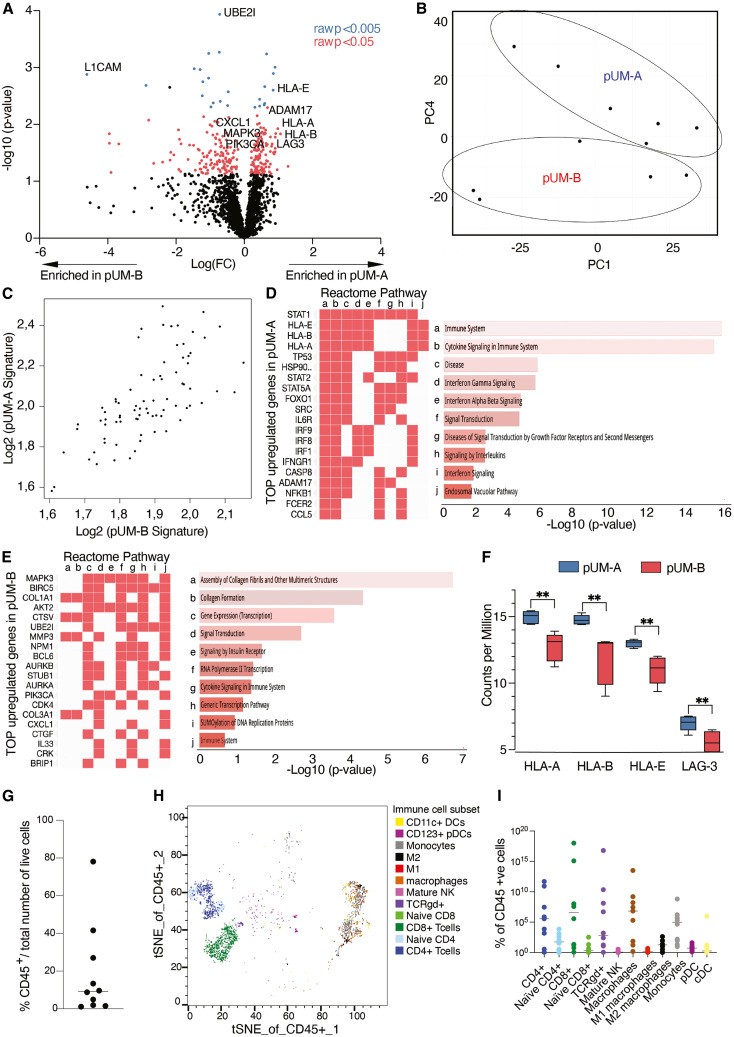
Molecular phenotype of primary uveal melanoma samples (A) Volcano plot showing differentially expressed genes between pUM-A and pUM-B. A negative log_2_-fold change indicates that the gene is enriched in pUM-B in comparison to pUM-A. Blue dots indicate highly significant associations (raw *p* < 0.005), and red dots denote significant (raw *p* < 0.05) association identified by explorative data analysis using Wilcoxon Mann-Whitney rank-sum test. (B) PCA plot showing the separation of samples based on expression profile within the two clusters pUM-A vs. pUM-B. Unit variance scaling is applied to gene counts. Singular value decomposition (SVD) with imputation is used to calculate principal components. *x* and *y* axes show principal component 3 and principal component 1 that explain 9.5% and 30.5% of the total variance, respectively. (C) Scatterplot (log-scale) of pairwise gene correlation analysis for gene expression data of pUM-A and pUM-B signatures within the TCGA dataset (UM) log_2_ values of gene signatures are indicated (*n* = 80). (D and E) Enrichment analysis of Reactome pathways on all significantly upregulated DEGs in pUM-A (D) and pUM-B (E) samples, defined as a raw *p* value < 0.05, Top 10 significantly upregulated pathways are shown together with the respective genes contributing to each pathway. (F) Boxplots of counts per million for the differentially expressed exemplary immunomodulative molecules in pUM-A vs. pUM-B (mean ± SD). Welch two-sample *t* test; ∗∗*p* ≤ 0.05. (G–I) Spectral flow immunophenotyping of a distinct group of *n* = 10 pUM samples showing the ratio of immune cell infiltration (G), a tSNE plot of immune cell subset distribution in a representative sample (H), and quantification across all pUM samples (I) (single values and lines at median are shown).

To get deeper insights into the suggested molecular phenotypes that distinguish pUM-A from pUM-B tumors, we further investigated the genetic profiles of both subsets. Comparing gene expression between pUM-A and pUM-B samples we identified a total of 176 significantly differentially expressed genes (DEGs) (Figure 2A). We found that 107 of 176 DEGs (61%) were overexpressed in pUM-A (*p* ≤ 0.05) compared with pUM-B, whereas 69 targets (39%) were increased in pUM-B (*p* ≤ 0.05). Further exploratory analysis showed a clear separation of pUM samples in principal-component analysis (PCA) plots, which could be correlated with the two molecular subsets previously identified by hierarchical clustering (Figure 2B).^15^ To further test the robustness of our identified gene signatures, we employed gene expression profiling interactive analysis (GEPIA2) to investigate the expression levels of selected key genes from pUM-A and pUM-B samples in a larger UM cohort. The underlying dataset was derived from a comprehensive multiplatform analysis of 80 UMs.^9^ The resulting pairwise correlation analysis of gene expression data for pUM-A and pUM-B signatures within The Cancer Genome Atlas (TCGA) dataset showed robust concordance with our dataset, as illustrated in the scatterplot (*p* = 8.1e−11, R = 0.65) (Figure 2C).

To further classify the identified subgroups of pUM based on their gene expression profiles, we performed a gene set enrichment analysis (GSEA). Interestingly, Reactome pathway analysis revealed an overall inflammatory phenotype for the pUM-A subset, with significant upregulation of genes related to “immune system,” “interferon gamma signaling,” and “cytokine signaling” pathways (Figure 2D). Genes upregulated in pUM-B tumors exhibited a proliferative, desmoplastic phenotype, with “collagen formation” and “transcriptional and signal transduction” as significantly, dominating pathways (Figure 2E). Strikingly, the inflammatory phenotype of pUM-A tumors was associated with a significant increase in genes related to immunomodulating properties as HLA-A, HLA-B, HLA-E, and LAG3 when compared with pUM-B tumors (Figure 2F).^16^^,^^17^ Taken together, the clinically rather aggressive phenotype pUM-A not only showed frequent chromosomal aberrations but was also correlated with an inflammatory phenotype. To support our findings regarding the variability of immune infiltration in pUM tumors, we explored a separate sample set of single-cell suspensions from ten patients and performed a comprehensive immunophenotyping (IPT) via spectral flow cytometry (Figures 2G–2I). Strikingly, we found a high variation of immune infiltration across the samples. While various immune cell subtypes were identified within pUMs, our independent dataset was consistent with the transcriptomic profile of a predominantly adaptive immune infiltration.

### Metastatic lesions reveal (immune) phenotypic diversification compared to their primary counterparts

**Figure 3:**
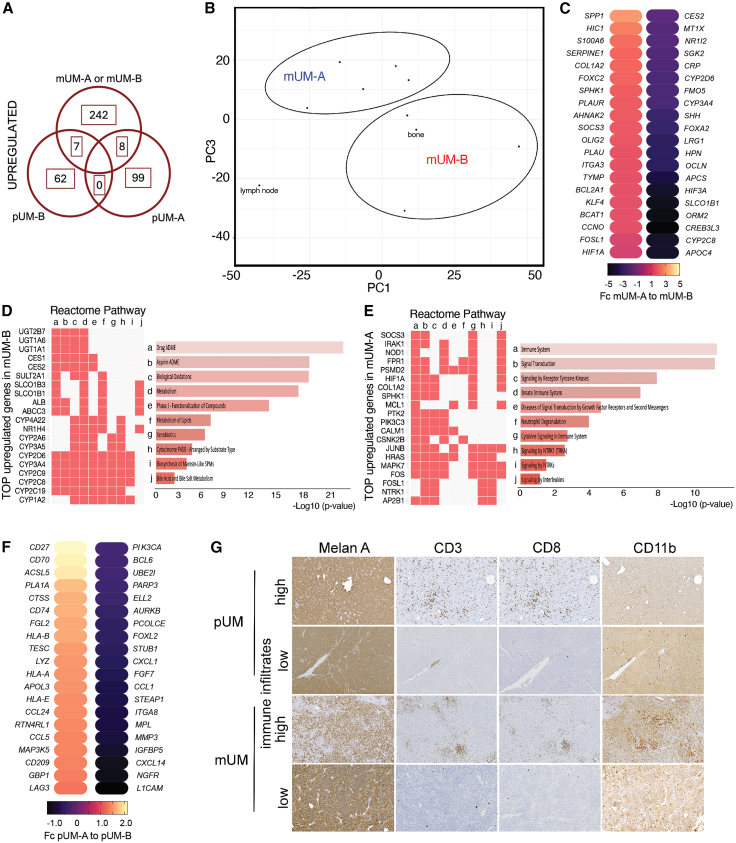
Low genetic overlap in primary and metastatic uveal melanoma samples (A) Venn diagrams illustrating the number of overlapping significant DEGs in pUM-A, pUM-B, and mUM samples. (B) PCA plot showing the separation of samples based on expression profile within the two clusters mUM-A and mUM-B. Unit variance scaling was applied to gene counts. SVD with imputation was used to calculate principal components. (C) Heatmap of top 20 upregulated or downregulated DEGs in mUM samples. DEGs have been defined as fold change (FC) > 1 or < −2 and raw *p* value < 0.05. (D and E) Enrichment analysis of Reactome pathways on all significantly upregulated DEGs (*n* = 149) in mUM-B samples (D) and all significantly upregulated DEGs (*n* = 108) in mUM-A samples (E), defined as a raw *p* value < 0.05. Top upregulated pathways are shown together with the respective genes contributing to each pathway. (F) Heatmap of top 20 upregulated or downregulated DEGs in pUM samples. DEGs have been defined as FC > 1 or < −0.5 and a raw *p* value < 0.05. (G) Representative immunohistochemistry images from pUM (*n* = 10) and mUM (*n* = 10) samples with indicated antibodies, from samples with high or low immune infiltration, respectively.

Next, we sought to further characterize the molecular phenotypes of matched metastatic lesions. The independent hierarchical classification identified two distinct molecular signatures, mUM-A and mUM-B (Figure 3E). To identify whether any overlap between primary and metastatic gene signatures existed in our examined samples, we performed a direct comparison of DEGs, as represented in the Venn diagram (Figure 3A). Even with the sum of all significantly upregulated genes in mUM-A and mUM-B, we found an overlap of only 8 genes with pUM-A (*ABCB4*, *ALDH4A1*, *CCL14*, *CCNO*, *CNTF*, *MGST1*, *PCSK6*, and *SHMT1*) and 7 genes with pUM-B (*ANPEP*, *FTH1*, *NGFR*, *NPM1*, *PARP3*, *SFRP2*, and *SMAD9*). These striking differences suggested a highly distinct molecular biological phenotype of metastatic lesions compared with their primary counterparts, thereby indicating phenotypic plasticity during metastasis formation. Further exploratory analysis of the processed sequencing data from metastatic samples showed a clear separation of the two mUM phenotypes and again revealed the lymph node metastasis as a separate entity with a unique transcriptomic profile compared with metastases of other organs (Figure 3B) as unsupervised hierarchical clustering had indicated (Figure 1E). Transcriptomics of the bone metastatic sample aligned well with the mUM-B profile.

We detected a total of 257 DEGs. We found that 108 of 257 (42%) DEGs were significantly overexpressed in mUM-A (*p* ≤ 0.05) compared to mUM-B samples, whereas 149 (58%) targets showed overexpression in mUM-B (*p* ≤ 0.05).

First, we identified the top 20 DEGs that were upregulated in either mUM-A or mUM-B (downregulated in mUM-A). The most upregulated genes in the mUM-B subtype indicated a metabolic phenotype including diverse CYP genes and the genes involved in lipid metabolism, such as *APOC4* (Figure 3C). In direct comparison, the mUM-A subtype was enriched for genes that promote cell survival and growth during cellular stress responses, such as *HIF1A* or *BCL2A1*, including DNA repair mechanisms (e.g., *KLF4*) as well as cell growth and proliferation (e.g., *ITGA3*, *SPHK1*, and *FOSL*) (Figure 3C). To confirm this notion, we implemented GSEA for all genes upregulated in mUM-B (Figure 3D) or mUM-A (Figure 3E). This unbiased approach confirmed an enrichment of Reactome pathways for metabolism and biologic oxidation in mUM-B, while pathways resembling signal transduction and immune system were predominantly present in mUM-A.

However, genes annotated as immune system related for mUM-A were mostly representative of the tumor-intrinsic properties that promote cell survival and growth under inflammatory pressure (Figure 3E). These included genes involved in early immune responses, including innate sensing, cytokine production, and inflammatory gene expression (e.g., *SOCS1*, *IRAK1*, and *NOD1)* as well as genes associated with immune evasion (e.g., *FOSL1* and *SPHK1*). This predominantly innate inflammatory phenotype present in mUM-A was very distinct to the immune phenotype that was evident in pUMs of subtype pUM-A. Analysis of the top DEGs upregulated in pUM-A compared to their spatial counterpart pUM-B revealed a strong emphasis on adaptive immune activation in pUM-A, dominated by genes involved in immune activation, signaling, or regulation (e.g., *CD27*, *CD70*, *CTSS*, *CD74*, *CCL5*, and *CD209*) and indicating immune surveillance (e.g., *HLA-A*, *HLA-B*, and *HLA-E*), but which might possibly be constrained by exhaustion or immune evasion mechanisms, such as *LAG3* (Figure 3F).

Conclusively, the pUM-A subtype describes an inflammatory immune-evasive phenotype in the context of adaptive immune responses, while the mUM-A cluster is related to inflammation-associated, tumor-intrinsic stress responses connected to early innate immune reactions. We further validated our findings of variable immune infiltration in an independent cohort of 20 unmatched samples (pUM: *n* = 10; mUM, *n* = 10; liver). In line with the IPT of pUMs (Figure 2G), immunohistochemistry of the pUMs revealed a high variability in the amount of immune infiltration across samples (Figure 3G). These samples were not transcriptionally characterized and were exclusively used to assess innate versus adaptive immune dominance at the cellular level, as suggested by our transcriptomic findings. Histopathological analysis of liver metastases showed a replacement pattern of mUMs. The variability of overall immune infiltration as in pUM samples was similarly observed in mUM samples, with a tendency toward increased innate immune cell infiltrates in liver metastases (CD11b) compared with adaptive immune cells (CD3 and CD8) in central tumor regions of pUM samples.

### Gene signature of pUMs correlates with OS in metastatic disease

**Figure 4:**
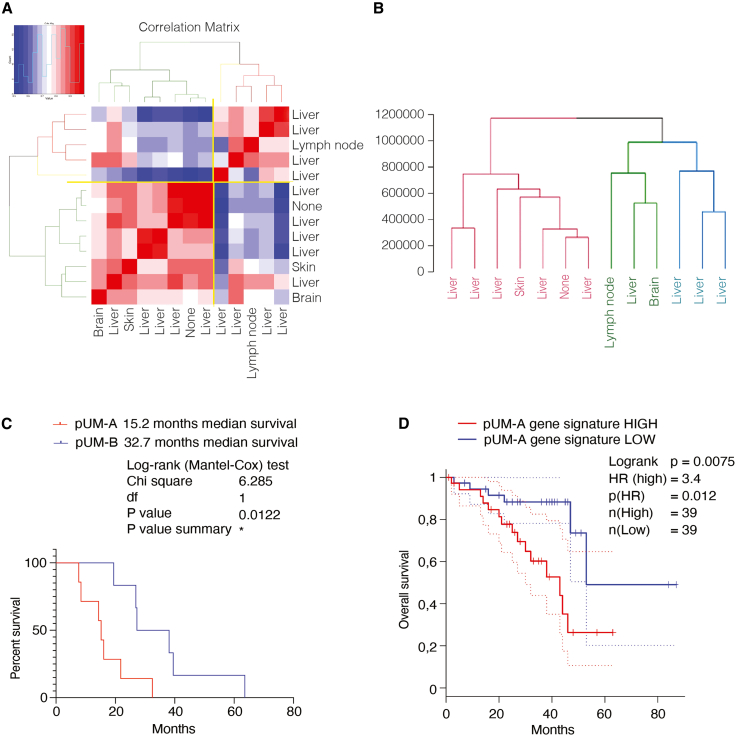
Association of biological phenotype of the primary tumor with the localization of metastasis and survival prediction (A) Heatmap clusters for the individual primary tumor samples (pUM-A vs. pUM-B) tagged by first diagnosed metastatic localization (dark color corresponds to parity between both samples). (B) Unsupervised hierarchical clustering of biological phenotype of the primary tumor tagged by first diagnosis of metastatic localization (no pUM patient died before diagnosis of metastatic lesion). The *y* axis depicts the height of the branches, indicating the dissimilarity between clusters. (C) Kaplan-Mayer estimate: patients’ survival after diagnosis of liver metastasis depending on molecular biological phenotype of the primary tumor pUM-A vs. pUM-B. (D) Kaplan-Meier survival curve of patients with gene expression data of our primary tumor (pUM-A) signatures validated with the TCGA database for UM cohort (*n* = 78). Hazard ratio (HR) and *p* value are indicated.

Subsequently, we aimed to assess the predictive value of pUM subtypes with respect to the site of metastasis and overall patient survival. First, we investigated whether the identified biological phenotype of the primary tumors pUM-A and pUM-B correlated with the location of metastasis as presented in the heatmap (Figure 4A). One patient died before the detection of metastasis and was, therefore, classified as “none.” Unsupervised clustering of the examined genes in relation to first diagnosed metastatic manifestation of the pUM patients did not result in a clear pattern for extrahepatic metastases (Figure 4B). However, conclusions of this correlative analysis are limited due to the small sample size.

We then performed a Kaplan-Meier analysis to evaluate if the molecular profile of the primary tumor correlates with OS. Strikingly, pUM-B subgroup patients displayed better OS after the diagnosis of metastatic disease than the pUM-A subgroup patients (median OS: 15.1 vs. 32.7 months), with a log-rank (Mantel-Cox) test showing a chi-square value of 6,285 and *p* value of 0.0122 (Figure 4C). This observation was in line with the identified more aggressive phenotypic traits of pUM-A compared with those of pUM-B. To confirm the robustness of our results, we evaluated the predictive value of the pUM-A signature for OS, using the independent TCGA dataset. Patients whose primary tumors were characterized by a high expression of the inflammatory immune-evasive phenotype pUM-A exhibited significantly worse survival (HR = 3.4 and *p* = 0.0075) (Figure 4D).

### Matched molecular phenotype profiling indicates phenotypic dissociation of metastatic lesions from primary tumors

**Figure 5:**
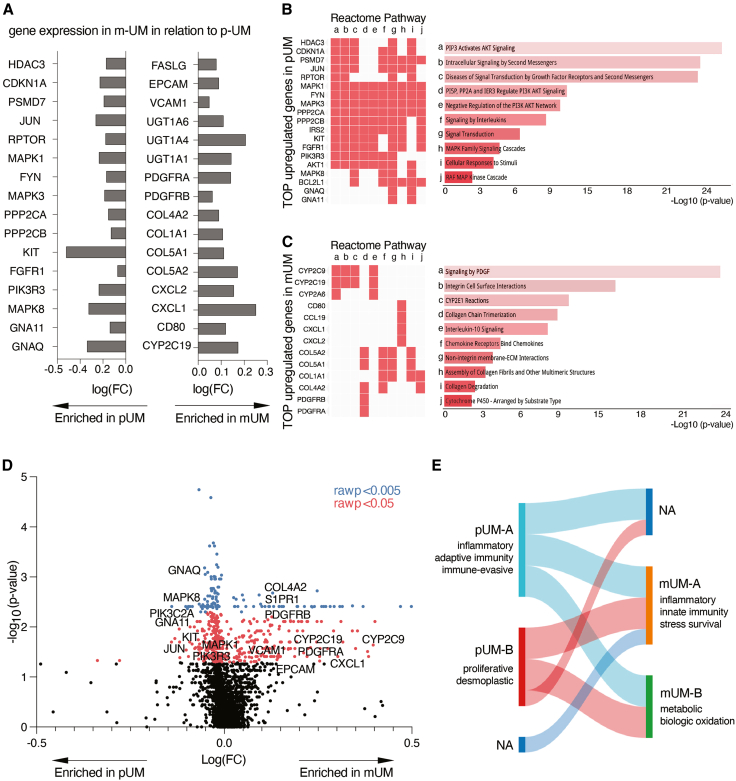
Association of biological molecular phenotype of pUMs with matched mUMs (A) Bar chart of top Reactome-classified DEGs from 335 upregulated genes in primary tumors and 217 downregulated DEGs comparing pUM to mUM. DEGs have been defined as genes with a raw *p* value < 0.05. (B and C) Enrichment analysis of Reactome pathways on all significantly upregulated DEGs in all matched pUM to mUM samples (pUM/mUM) (B) or mUM to pUM samples (mUM/pUM) (C), defined by raw *p* value < 0.05. Top 10 significantly upregulated pathways are shown together with the respective genes contributing to each pathway. (D) Volcano plot showing the statistical significance and fold change (FC) of DEGs between pairs of mUM/pUM, measured using an expression ratio. A negative log_2_ FC indicates that the gene is upregulated in the primary samples in comparison to the metastatic samples. Blue dots indicate highly significant associations (raw *p* < 0.005), and red dots denote significant (raw *p* < 0.05) associations identified by explorative data analysis using Wilcoxon Mann-Whitney rank-sum test. (E) Sankey plot of the observed molecular biological phenotypes (gene signatures) throughout metastasis: pUM-A: 6 inflammatory, adaptive immunity, and immune evasive phenotype samples; 5 pUM-B: proliferative and desmoplastic phenotype samples. Metastasis: 5 mUM-A, inflammatory, innate immunity, and stress survival phenotype samples; 4 mUM-B, metabolic and biologic oxidation phenotype samples; NA: not able to specify to one of the respective groups. Left: 1 pUM with insufficient transcriptomic data partnered with an mUM-A sample. Right: samples originating from two pUM-A samples as well as one pUM-B sample with missing partner metastasis.

Finally, to investigate molecular plasticity through the process of metastasis, we chose to compare overall significant changes in gene expression of the matched cohorts of mUM samples to primary tumors. We found that 552 of 2,549 genes showed a significantly altered expression. Among these, 335 genes were upregulated in pUM (*p* ≤ 0.05), while 217 were upregulated in the matched mUM samples (*p* ≤ 0.05). *GNAQ*, *GNA11*, as well as genes involved in downstream signaling pathways of mitogenic signaling and proliferation, including *PIP3*, *KIT*, *PPP2CA*, *JUN*, *PIK3R3*, *MAPK1*, *MAPK3*, and *MAPK8*, were among the dominant upregulated genes in pUM (Figures 5A, 5B, and 5D). Because the principal drivers of UMs are the mutations in either *GNAQ* or *GNA11*, which account for about 95% of all UMs, it is not surprising that these pathways were significantly upregulated in primary tumors. However, the reduced significance of these gene expression patterns in metastatic lesions was remarkable. In contrast, DEGs identified in mUMs indicated a pro-tumoral inflammatory microenvironment (e.g., *CXCL1* and *CXCL2*) combined with a desmoplastic tumor phenotype (e.g., *PDGFRA*, *PDGFRB*, *VCAM1*, *EPCAM*, and *VCAN)* and markers of metabolic activity (e.g., *COL5A1*, *COL5A2*, *CYP3A*, *CYP2C8*, *CYP3A4*, and *CYP2A6)* (Figures 5A, 5C, and 5D).

Because our gene expression analysis revealed overall significant changes between pUMs and metastases, we aimed to determine if and to what extent subtype-specific phenotypes were maintained during metastasis. Thus, using our matched primary and metastatic tumor pairs, we tested if the pUM subtype predetermined phenotype switching into a particular mUM subgroup. To this end, we generated a Sankey association plot (Figure 5E). The illustration depicts the transition of the identified primary tumor phenotypes throughout the metastasis formation toward the metastatic phenotypes. There was no obvious association of pUM subtypes with a specific metastatic phenotype. Primary tumors with a predominant inflammatory signature (pUM-A) gave rise to inflammatory signature metastasis (mUM-A) only in half of the cases. The remaining half transitioned from the inflammatory pUM-A phenotype to the metabolic, oxidative phenotype mUM-B. Similarly, half of the cases with desmoplastic, proliferative phenotype (pUM-B) samples transitioned to the mUM-A metastatic inflammatory phenotype, and others showed a phenotype switching to the metabolic, oxidative phenotype (mUM-B) during metastasis formation. Taken together, our data strongly suggest that the pUM subtype does not predetermine a specific mUM phenotype. Thus, during metastasis formation in UM phenotypic identities change in a fashion that is uncoupled between primary and metastatic tumors.

## Discussion

Our work sheds light on divergent phenotypic profiles of pUMs and mUMs. It also provides evidence that pUM subtypes are not always related to the phenotypes of their respective matched mUM counterparts. Previous studies have demonstrated the genetic and epigenetic evolution of UM by analyzing matched samples before and after metastatic spreading.^18^^,^^19^ However, to the best of our knowledge, this is the first comprehensive analysis of gene expression profiles of primary tumors in relation to matched metastases, assessing changes in the transcriptomic molecular phenotype.

We identified two main subtypes for each entity, both in primary and metastatic tumors. Strikingly, while almost all UM cases are driven by mutation in either *GNAQ* or *GNA11*^20^^,^^21^ only approximately half of the pUM tumors showed a dominant MAPK/PI3K-signaling-addicted proliferative and desmoplastic tumor phenotype. This observation raises questions regarding the efficacy of currently developed therapeutic strategies targeting PKC. Clinical trials on pUMs with the PKC inhibitor darovasertib have shown very promising results with local tumor control in almost every second patient.^22^^,^^23^ Yet, the underlying pathophysiological dependencies and resulting alternative strategies for the remaining non-responders remain unclear. Preclinical research suggests diverse synthetic and synergistic mechanisms that could be harnessed for biologically informed combination therapies. For instance, a recent study identified inositol polyphosphate-5-phosphatase A (INPP5A) as a synthetic lethal target in mutant melanoma cell lines.^24^ Moreover, co-targeting of the PI3K/AKT or MAPK pathways can synergize with PKC inhibitors.^25^^,^^26^ Inhibition of the MAPK pathway alone has already proven clinically beneficial as it led to an increase in progression-free survival (PFS) in mUM patients^27^ and might further support PKC inhibitor-based treatments.

However, our data indicate that an immunomodulating therapeutic approach could be beneficial to a subset of patients with UM, namely the inflamed subtype pUM-A. Despite the assignment of the eye as an “immune-privileged” site,^28^ our data as well as unrelated published data^29^ provide evidence that immune infiltration into pUMs can take place. We show that this inflamed, immune-evasive subtype is related to a particularly aggressive phenotype with worse overall patient survival (pUM-A), which was further validated in the TCGA dataset. This is in line with current findings that chromosomal aberrations in UMs are associated with an inflammatory microenvironment and progressive disease.^30^^,^^31^ Further, increased expression of the T cell inhibitory immune checkpoint LAG has been discovered in high-risk pUMs.^32^ In line with this finding, we confirmed the altered LAG-3 levels in M3 patients within our observed cohort. Taken together, these data indicate that patients with the pUM-A subtype might benefit from neoadjuvant or adjuvant (combinational) immunotherapies. However, due to phenotypic plasticity, additional studies in larger patient cohorts are required to draw conclusions on the reliability and predictive value of immune subtypes.^33^^,^^34^

Notably, an inflammatory subtype was identified in both pUM (designated as pUM-A) and mUM (designated as mUM-A) tumors. Yet, their immune signatures displayed markedly distinct features. The inflammatory subtype of pUM was dominated by an adaptive immune response with IFN signaling and an immune evasive profile including increased LAG3 expression. In stark contrast, the inflammatory subtype of mUM reflected strong early innate immunity signaling and the activation of tumor-intrinsic stress and survival pathways. In line with this finding, it has been observed that immune infiltrates in UM liver metastases exhibit a pro-tumorigenic M2 phenotype of tumor-infiltrating macrophages.^35^ Another study demonstrated that BAP1 loss in mUM was associated with an immunosuppressive phenotype, with increased expressions of IDO1 and PD-L1.^36^ The immunotherapeutic targets of primary and metastatic UMs may differ significantly, and, therefore, future clinical trials on mUM treatment should involve therapeutic modulation of the innate immune pathways.^36^ Previous subgroup analyses of UM patients treated with the immune checkpoint inhibitors ipilimumab and nivolumab revealed a statistical benefit only for patients with extrahepatic metastasis.^37^^,^^38^^,^^39^ Yet, it should be mentioned that *ex vivo* expansion and adoptive transfer of autologous tumor-infiltrating lymphocytes (TILs) from UM patients has been shown to mediate regression of metastatic UMs.^40^ This finding further demonstrates that tumor-reactive T cells are present even in liver metastases. However, the local immunosuppressive environment may promote immune evasion, which might be overcome by extensive delivery of *ex vivo* (re-)activated TILs.

Moreover, the direct gene expression comparison between matched pUM versus mUM cohorts revealed a statistically significant increase in secondary messenger pathways that are associated with enhanced survival and proliferation only in pre-metastatic samples. Instead, most significant gene signatures for mUM were related to cell adhesion, modulation of the extracellular matrix, and immunosuppression (e.g., IL-10 signaling). Again, these findings underline the context-dependent phenotypic identities of cancer cells. Accordingly, therapeutic adjustments must be applied in a manner that takes the phenotypic profile and the metastatic sites into account. For instance, the ongoing clinical trial with the PKC inhibitor IDE196 is combining therapy with the tyrosine kinase inhibitor crizotinib in mUM (NCT05987332).

Personalized cancer profiling of pre-metastatic disease might help guide clinical decision-making to enable therapeutic success with individualized treatments. Loss of one copy of chromosome 3 is associated with worse OS in patients with metastatic melanomas^41^ and has been associated with the inactivation of MHC structural genes in UMs.^42^ Herein, we identified distinct immunogenic and non-immunogenic phenotypes of late-stage mUM. Yet, pUM immunogenicity did not predict mUM immunogenicity.

Metastasis is a multistage process that includes challenges for cancer cells, particularly in terms of survival, adaptation, and replication in a new environment.^43^^,^^44^ It had been proposed that pUM cells might transfer their immune-privileged properties to metastatic sites.^28^ However, our data strongly suggest that the molecular phenotype of primary tumors is not predictive for metastatic tumor subsets. Instead, the (immune) phenotypes of established tumors are specific to the target organ.^45^ Studies on other cancer entities have further demonstrated that clonal diversity and tumor evolution, even in early stages of the disease, lay the foundation for later resistance and metastasis.^46^^,^^47^

### Limitations of the study

While this work provides novel insights into the divergent transcriptomic phenotypes of matched pUM and mUM samples, the limited sample size of matched pairs should be acknowledged. This constrains the statistical power and the generalizability of subtype classification. Yet, we included IPT and immunohistochemistry of independent patient cohorts, which support our findings concerning immunogenicity of the subclasses of pUM and mUM, as well as immune-phenotypic diversity. Taken together, our data delineate robust transcriptomic patterns that form a foundation for future functional studies and clinical validation in larger, prospectively collected cohorts.

## Resource availability

### Lead contact

Requests for further information, resources, and reagents should be directed to and will be fulfilled by the lead contact, Halime Kalkavan (halime.kalkavan@uk-essen.de).

### Materials availability

This study did not generate new unique reagents.

### Data and code availability

• All data supporting findings of this study are provided within the article and its supplemental information section. The transcriptomic datasets generated within the current study are available in the GEO repository, with the accession code GEO: GSE317536 (RNA sequencing data).
• This paper does not report original code.
• Any additional information required to reanalyze the data reported in this paper is available from the lead contact upon reasonable request.

## Acknowledgments

This work was supported by grants from the Advanced Clinician Scientist Programm UMEA^2^ (Medical Faculty, University Duisburg-Essen, the Federal Ministry of Education and Research 01EO2104 BMFTR), and the German Cancer Aid (Max-Eder Junior Research Group Program, DKH 70115382) to H.K. As indicated, some of the results published here are based on data generated by the TCGA Research Network: https://www.cancer.gov/tcga.

## Author contributions

Conceptualization, K.A.-G., S.B., M.W., F.M., and H.K.; data curation, K.A.-G., S.B., S.L., A.R.A.K., F.-Y.C., G.A., T.B., A.J., S.H., O.D.M., L.J., M.Z., T.K., K.K., and H.K.; formal analysis, K.A.-G., A.R.A.K., S.L., and H.K.; investigation, K.A.-G., A.R.A.K., S.L., U.B.-P., F.M., and H.K.; methodology, S.B., S.L., A.R.A.K., F.-Y.C., G.A., M.Z., M.W., and H.K.; software, K.A.-G., A.R.A.K., S.L., and F.M.; validation, S.L., A.R.A.K., F.-Y.C., G.A., and M.W.; visualization, K.A.-G., S.L., A.R.A.K., and M.W.; writing – original draft, K.A.-G., M.F., U.B.-P., and H.K.; writing – review & editing, K.A.-G., S.B., T.B., A.J., O.D.M., M.Z., M.W., M.F., L.J., U.B.-P., N.B., and H.K.; funding acquisition, K.A.-G., M.F., N.B., F.M., and H.K.; resources, T.B., A.J., S.H., O.D.M., T.K., L.J., K.K., U.B.-P., N.B., F.M., and H.K.; project administration, K.A.-G., F.M., and H.K.; supervision, N.B., F.M., and H.K.

## Declaration of interests

The authors have no competing interests to disclose for all submitted content.

## STAR★Methods

### Key resources table

**Table:** 

REAGENT or RESOURCE	SOURCE	IDENTIFIER
**Antibodies**		

CD11b antiAnti-CD11b antibody [EPR1344]	Abcam	Cat# ab133357; RRID:AB_2650514
Anti-CD3 epsilon antibody [SP7]	Abcam	Cat# ab16669; RRID:AB_443425
Anti-CD8 alpha antibody [SP16]	Abcam	Cat# ab101500; RRID:AB_10710024
Melan-A Polyclonal antibody	Proteintech	Cat# 18472-1-AP; RRID:AB_2878545
Ki67	Roche	Cat# 790–4286; RRID:AB_2631262
Anti-Human CD197 (CCR7)-BV421, Clone: G043H7	Biolegend	Cat# 353207, RRID: AB_10915137
Anti-Human CD3-BV570, Clone: UCHT1	Biolegend	Cat# 300435, RRID: AB_10898117
Anti-Human CD28-BV650, Clone: CD28.2	Biolegend	Cat# 302945, RRID: AB_2616854
Anti-Human CD56 (NCAM)-BV750, Clone: 5.1H11	Biolegend	Cat# 362555, RRID: AB_2734396
Anti-Human CD279 (PD-1)-BV785, Clone: EH12.2H7	Biolegend	Cat# 329929, RRID: AB_11218984
Anti-human CD11c-PE, Clone: 3.9	Biolegend	Cat# 301605, RRID: AB_314175
Anti-Human CD38-BV711, Clone: HIT2	Biolegend	Cat# 303527, RRID: AB_11218990
Anti-Human IgM-BV510, Clone: MHM-88	Biolegend	Cat# 314521, RRID: AB_2561513
Anti-Human CD206 (MMR)-AF488, Clone: 15-2	Biolegend	Cat# 321113, RRID: AB_571874
Anti-Human CD68-AF647, Clone: Y1/82 A	Biolegend	Cat# 333819, RRID: AB_2571962
Anti-Human CD45R (B220)-BUV496, Clone: RA3-6B2	Thermo Fisher Scientific	Cat# 364-0452-80, RRID: AB_2920961
Anti-Human CD80 (B7-1)-BUV661, Clone: 2D10.4	Thermo Fisher Scientific	Cat# 376-0809-41, RRID: AB_2925457
Anti-Human CD86 (B7-2)-SB600, Clone: IT2.2	Thermo Fisher Scientific	Cat# 63-0869-41, AB_2717048
Anti-Human RON (CD136)-BUV395, Clone: Zt/g4	BD Biosciences	Cat# 745593, AB_2743102
Anti-Human CD8-cFluor B532, Clone: SK1	Cytek Biosciences	Cat# R7-20124
Anti-Human CD14-cFluor B548, Clone: 63D3	Cytek Biosciences	Cat# R7-20116
Anti-Human HLA-DR-cFluor B690, Clone: L243	Cytek Biosciences	Cat# R7-20126
Anti-Human CD4-cFluor YG584,Clone: SK3	Cytek Biosciences	Cat# R7-20042
Anti-Human CD16-cFluor V450, Clone: 3G8	Cytek Biosciences	Cat# R7-20184
Anti-Human IgD-cFluor BYG667, Clone: IA6-2	Cytek Biosciences	Cat# R7-20138
Anti-Human TCRγδ-cFluor BYG710, Clone: B1	Cytek Biosciences	Cat# R7-20136
Anti-Human CD127-cFluor R659, Clone: eBioRDR5	Cytek Biosciences	Cat# R7-20466
Anti-Human CD19-cFluor R685, Clone: HIB19	Cytek Biosciences	Cat# R7-20118
Anti-Human CD123-cFluor R720, Clone: 6H6	Cytek Biosciences	Cat# R7-20014
Anti-Human CD45-cFluor R780, Clone: 2D1	Cytek Biosciences	Cat# R7-20134
Anti-Human CD27-cFluor R840, Clone: QA17A18	Cytek Biosciences	Cat# R7-20082
Anti-Human CD15-cFluor V505, Clone: W6D3	Cytek Biosciences	Cat# R7-20348
Anti-Human CD11b-cFluor BYG610, Clone: ICRF44	Cytek Biosciences	Cat# R7-20336
Anti-Human CD33-cFluor BYG781, Clone: WM53	Cytek Biosciences	Cat# R7-20342

**Reagents**		

CC1-buffer	Roche	Cat# 950-124
ViaDye™ Red Fixable Viability Dye	Cytek Biosciences	Cat# R7-60008
DNAse I	Sigma-Aldrich	Cat# DN25
Collagenase I	Thermo Fisher	Cat# 17100017
Collagenase II	Thermo Fisher	Cat# 17101015
Hydrogen peroxide	Carl Roth	Cat# 1A8Y.1
Hydrogen peroxide	Carl Roth	Cat# 7722-84-1
Antigen Unmasking Solution, Citrate-Based	Vector Laboratories	Cat# H-3300-250
Antigen Unmasking Solution, Tris-Based	Vector Laboratories	Cat# H-3301-250
Blocking Solution	Zytomed Systems	Cat# ZUC007-100
SignalStain® Antibody Diluent	Cell Signaling Technology	Cat# 8112 L
ZytoChem Plus (HRP) One-Step Polymer anti-Mouse/Rabbit	Zytomed Systems	Cat# ZUC053-100
Dako Liquid DAB+ Substrate Chromogen System	Agilent Technologies	Cat# K3468
Mayer’s hemalumsoluton	Sigma-Aldrich	Cat# 1.09249.0500
Pertex® Histolab Mounting Medium	Histolab	Cat# 00811-EX
Kapa Library Quantification	Roche	Cat# 07960140001

**Critical commercial assays**		

HTG Transcriptome Panel HTG	HTG Molecular diagnostics	https://www.htgmolecular.com/

**Software and algorithms**		

HTG EdgeSeq Reveal Software (v5.4.0.7543)	HTG Molecular Diagnostics	https://www.htgmolecular.com/
R (v4.0.3)	The R Fondation	https://www.r-project.org/
Illumina NextSeq 500	Illumina	https://emea.illumina.com
Prism (10.6.1)	Graphpad	https://www.Graphpad.com/
FlowJo software	Tree Star	N/A
WEB-based Gene SeT AnaLysis	WebGestalt	https://www.webgestalt.org
enrichR	Wajid Jawaid	SCR_001575
GEPIA2	Zefang Tang	SCR_026154
cTree	Torsten Hothorn	https://doi.org/10.1145/1031453.1031462
edgeR	Yunshun Chen	https://doi.org/10.18129/B9.bioc.edgeR
pcaMethods R	Tauno Metsalu	https://doi.org/10.1093/nar/gkv468

**Other**		

Deposited RNA Raw data	This Paper	NCBI GEO: GSE317536

### Experimental model and study participant details

This retrospective study included samples from 53 patients (24 male, 19 female). Samples were obtained in accordance with the ethical guidelines and approval of the Ethics Committee of the University of Duisburg-Essen (24-12242-BO), with informed consent provided by the patients. Information regarding the ancestry, race, or ethnicity of the participants was not routinely collected in the clinical databases used for this retrospective cohort and is therefore not reported.

#### Patients and samples

The study design followed a discovery-oriented workflow with supportive analyses in independent cohorts (Figure S1). For initial transcriptomic profiling, a discovery cohort of 23 treatment-naive samples was used, comprising 13 enucleated pUM and 10 mUM samples. To investigate phenotypic evolution during progression, comparative and phenotype-switching analyses were restricted to the 10 patients with matched pUM and mUM samples.

To further support the transcriptomic findings regarding the variability of immune infiltration, two independent cohorts were analyzed: (a) single-cell suspensions from 10 independent pUM patients (not included in the transcriptomic discovery cohort) were assessed by spectral flow cytometry, and (b) an additional cohort of 20 unmatched samples (10 pUM and 10 mUM) was analyzed by immunohistochemistry to characterize innate versus adaptive immune cell predominance. These independent samples were not transcriptionally characterized and were used exclusively for orthogonal cellular-level assessment of immune infiltration patterns suggested by the discovery cohort.

#### Treatments and evaluation of matched samples patient cohort

Patients were treated at the discretion of their physician. Routine follow-up was similar across the two cohorts included in the study. Progress free survival (PFS) was defined as the time from the start of treatment until disease progression, death, or last patient contact. OS was defined as the time from the start of treatment until death or last patient contact. For initial transcriptomic profiling, 13 pUM samples were analyzed via unsupervised hierarchical clustering to ensure a robust classification of primary molecular subtypes. To maintain the core focus of the study on phenotypic evolution during progression, subsequent comparative analyses between primary and metastatic sites were restricted to the 10 patients with matched pUM and mUM samples. Tumor volume in primary tumors was assessed on enucleated tumor samples, if not available using pre-operative ultrasound measurement. The study protocol is in accordance with the Declaration of Helsinki and approved by the Ethics Commission of the University of Essen (ref. 24-12242-BO).

### Method details

#### Immunohistochemistry

Tissue was formalin fixed, paraffin embedded and sectioned at 4 μm thickness before dewaxing. For Ki67-proliferation index staining reactions were carried out with a Ventana Autostainer (Roche, Mannheim, Germany). The following antibodies were used: Ki-67 (clone 30–9; Roche, Mannheim, Germany), concentration 2 μg/mL, antigen retrieval for 32 min, 95°C, CC1-buffer (Roche, Mannheim, Germany, CC1-buffer (Roche, Mannheim, Germany). The immunohistochemical Ki67 stain was assessed by evaluating the nuclear staining response of tumor cells. The results were reported in % of positive tumor cell nuclei.

All other stainings were performed manually. Tissue sections, from core biopsies of liver metastases or enucleated eyes respectively, were deparaffinized in xylene and rehydrated through a graded series of ethanol (100%, 95%, 70%) to distilled water. Antigen retrieval was performed in a pressure cooker using pH 6 buffer for CD11b and MelanA, and pH 9 buffer for CD3 and CD8. Photobleaching was carried out five times, 45 min per cycle, to reduce background autofluorescence. After blocking endogenous peroxidase activity and nonspecific protein binding, slides were incubated with primary antibodies at 4 °C overnight. On the following day, slides were incubated with secondary antibodies at room temperature for 30 min. Antibody binding was visualized using an HRP-DAB detection system, followed by nuclear counterstaining with hematoxylin. Finally, tissue sections were dehydrated through a graded ethanol series (70%, 96%, 100%), cleared in xylene, and mounted with permanent mounting medium and coverslips for microscopic examination.

#### Immunophenotyping via spectral flow

Tumor single-cell suspension preparation: Excised tumors were collected, minced, and digested with collagenase I (1 mg/mL, Thermo Fisher), collagenase II (1 mg/mL, Thermo Fisher), and DNase I (50 μg/mL, Sigma-Aldrich) at 37 °C for 30 min with gentle agitation. Digested tissue was filtered through a 70 μm cell strainer, washed with PBS, and viable cells were counted.

Cell staining for flow cytometry: Approximately 200,000 cells from tumor single-cell suspensions were transferred into a U-bottom 96-well plate and washed with PBS. Cells were stained with 5 μL ViaDye Red Fixable Viability Dye in PBS (0.2% v/v, Cytek Biosciences) at room temperature for 15 min in the dark. After washing, cells were stained with a custom antibody panel (see Antibodies section for full list), prepared in FACS buffer (1 g/L sodium azide, 10 g/L PBS powder, 2.5 mM EDTA, 1% FCS in MilliQ-filtered water), for 20 min at room temperature protected from light. Cells were washed again, resuspended in 150 μL FACS buffer, and acquired on a Cytek Aurora 5-laser spectral analyzer. Data were analyzed using FlowJo software (Tree Star).

#### Mutational status

Microsatellite analysis from tumor DNA of 23 markers was performed for 3–4 on each arm of chromosomes 3, 6, and 8 to identify chromosomal abnormalities as previously described.^48^ Chromosome 3 alterations were classified as Monosomy 3 (M3), Disomy 3 (D3) and allelic imbalance (AI).

#### Transcriptomic analyses

RNA expression profiling was performed on formalin-fixed paraffin-embedded (FFPE) tissue using the HTG EdgeSeq Oncology Biomarker Panel (OBP; HTG Molecular, Tucson, United States), a targeted transcriptomic assay covering 2,549 human transcripts. In contrast to conventional whole-transcriptome RNA sequencing workflows, the HTG EdgeSeq system is based on an extraction-free nuclease protection assay (qNPA) and was used according to the manufacturer’s instructions. For sample preparation, approximately 11 mm^2^ of tumor tissue was macrodissected from the center of each sample using a 10 μm thick FFPE section. The protected probe–target hybrids were subsequently amplified and barcoded to generate sequencing libraries. Libraries were quantified by quantitative PCR using the Kapa Library Quantification Kit (Roche, Basel, Switzerland) and sequenced on an Illumina NextSeq 500/550 platform using the NextSeq High Output Kit v2.5 (75 cycles, single-end reads), with up to 24 libraries processed per run. FASTQ files were generated and processed using HTG EdgeSeq Parser software (v5.4; HTG Molecular), with read alignment performed against the HTG reference probe set using Bowtie. Overall sequencing quality was high, with 94.6% of bases achieving a Phred quality score ≥ Q30. Across 24 libraries, total read counts ranged from 1368 to 109.81 million reads per sample, with 1245 to 104.99 million aligned reads and alignment rates between 91.01% and 96.14%. Sample exclusion was based on predefined technical QC criteria provided by the assay workflow, primarily severe under-sequencing with non-interpretable expression output; only one sample met these exclusion criteria, resulting in a final dataset of 23 samples, with a minimum of 1.46 million reads per sample. Quality control, normalization, and downstream analyses were performed using the HTG EdgeSeq Reveal Application and the R statistical environment (v4.0.3). Gene count data were normalized using the DESeq2 median-ratio method to account for differences in library size. For downstream statistical analyses and visualization, normalized counts were additionally log2-transformed. Supplementary analyses were performed using Prism (v10.4.0; GraphPad Prism).

#### PCA-plot

To visualize separation in-between sample configuration of the observed genes, unit variance scaling is applied to gene counts Singular Value Decomposition (SVD) with imputation is used to calculate principal components. X and Y axis show principal components that explain a certain amount of the total variance respectively^15^ (Figures 2D and 2E).

#### Volcano-plot

Examined genes are plotted using the -log10(*p*-value) in relation to the expression ratio with log2(foldchange) as an indicator (Figure 2F: Two values are outside of the visualized plot; Figure 4A: 29 values are outside of the visualized plot). For LogFC in Primary samples: Fold-changes were determined by either mean or median differences in gene expression between tested groups. Mean was calculated for normally distributed data, otherwise median calculation was applied. The logFC in the volcano plot pUM/mUM: was calculated as the ratio of the difference between log mean expression of count information in all metastatic samples (logmeanmetastatic) to log mean expression in all primary samples (logmeanprimary) also: (logmeanmetastatic − logmeanprimary)/logmeanprimary.

#### Validation of gene expression and survival analysis

For Immune signature gene expression comparison between our pUM cohort and 80 independent UM samples we utilized RNA sequencing expression data from the TCGA and the GTEx projects which were analyzed by the Webtool GEPIA2.^49^ Same datasets and software were used to determine disease free survival and overall survival in dependence of HRI expression in various tumors estimated by Mantel-Cox test (Figures 3D and 3E).

### Quantification and statistical analysis

The statistical details of experiments are available in the figure legends, figures, and results. The ‘‘n’’ indicated in the figure legends refers to the number of sample size used in the experiment. Graphical and statistical analyses were performed using R (v4.0.3) and GraphPad Prism (v10.4.0). The Shapiro–Wilk test was applied to assess normality of data distribution. Depending on the results, either parametric tests (Student’s *t* test, ANOVA) or non-parametric tests (Wilcoxon rank-sum test, Kruskal–Wallis test) were used to evaluate group differences. For categorical variables, Fisher’s exact test was applied to 2 × 2 contingency tables, while Pearson’s Chi-squared test was used for larger tables. Correlations between continuous variables were assessed using Pearson’s product–moment correlation coefficient or Spearman’s rank correlation test, as appropriate. RNA expression data were analyzed using the HTG EdgeSeq Reveal platform within the DESeq2 framework. Count data were normalized using the DESeq2 median-ratio method to account for differences in library size. Differential gene expression analysis was performed using negative binomial generalized linear models as implemented in DESeq2, with statistical significance determined after multiple testing correction using the Benjamini–Hochberg false discovery rate (FDR) procedure. Prior to differential expression analysis, principal component analysis (PCA) was conducted as an exploratory quality control step to assess global sample relationships and detect potential technical outliers. Sample exclusion was based on predefined technical quality control criteria and sequencing performance metrics. Additional exploratory data analysis included mean–variance plots for quality assessment. Unsupervised and supervised clustering approaches, as well as PCA, were applied to identify sample- and gene-level patterns. Heatmaps (Figures 1B and C) and clustering analyses were based on log-transformed counts per million (log CPM). Gene set enrichment analysis (GSEA) was performed using the enrichR package in R.^50^ Pathway enrichment analysis (Figures 2D, 2E, 3D, 3E, 5B, and 5C) was conducted using the Reactome Pathways 2024 database to identify significantly altered biological processes. Only genes with a *p*-value <0.05 from differential expression analyses were included. Directionality of pathway alterations was determined using the normalized enrichment score (NES), and pathways with *p*-values <0.05 were considered significant. Group differences (Figures 3C, 3F, and 5A) in gene expression were visualized using −log2 fold changes based on mean or median expression values, depending on data distribution. Fold changes (Figures 2A and 5D) were calculated accordingly using mean values for normally distributed data and median values otherwise. Marker performance was evaluated using receiver operating characteristic (ROC) analysis, with internal validation performed via bootstrap resampling. Overall survival (Figures 4C and 4D) (OS) was analyzed using Cox proportional hazards models. Multivariable Cox regression analyses were conducted to estimate both unadjusted and adjusted effects of clinicopathological and genetic covariates. Hazard ratios (HRs), 95% confidence intervals (CIs), and *p*-values derived from Wald, score (log rank), and likelihood ratio tests were reported. Cutoff values for biomarker stratification were determined using conditional inference trees (CTree), implemented in the “party” package in R, with leave-one-out cross-validation. CTree is a non-parametric class of regression trees based on conditional inference procedures, suitable for handling various data types, including nominal, ordinal, continuous, and censored variables, as well as multivariate responses.
