# Supplementary material for: Matched sampling reveals uncoupled phenotypic plasticity during metastasis formation in uveal melanoma
Source: iScience. 2026 Jun 19;29(7):116397. doi: 10.1016/j.isci.2026.116397 (PMC13315734; doi:10.1016/j.isci.2026.116397)
Supplement: Document S1. Figure S1 and Table S1 [file mmc1.pdf]

iScience, Volume *29*

Supplemental information

Matched sampling reveals uncoupled phenotypic plasticity during metastasis formation in uveal melanoma

Karim Al-Ghazzawi, Sabrina Borchert, Siyang Liu, Amal R. Al Kadi, Fung-Yi Cheung, Georgia Antonopoulou, Tobias Blau, Andreas Junker, Leyla Jabbarli, Sylvia Hartmann, Olaf D. Muras, Michael Zeschnigk, Tobias Kiefer, Kathy Keyvani, Michael Wessolly, Miltiadis Fiorentzis, Utta Berchner-Pfannschmidt, Nikolaos Bechrakis, Fabian Mairinger, and Halime Kalkavan

Patient	Location of metastasis	Dimensions	Sample	Sample Aquisition
1	Liver	1,5 X 1,3 X 0,4 cm	FFPE	Laparotomy
2	Liver	3cm Punch Cylinder	FFPE	Laparotomy with Punch-Biopsy
3	Liver	2,0 X 1,8 X 0,2cm	FFPE	Laparotomy
4	Liver	1,8cm Punch cylinder	FFPE	Laparotomy with Punch-Biopsy
5	Lymphnode	5,0 X 3,5 X 3,5cm	FFPE	Extirpation
6	Liver	2cm Punch cylinder	FFPE	Laparotomy with Punch-Biopsy
7	Liver	2cm Punch cylinder	FFPE	Laparotomy with Punch-Biopsy
8	Bone	3,5 X 4 X 0,7cm	FFPE	Humerus Biopsy
9	Liver	1,1 X 0,9 X 0,4 cm	FFPE	Laparotomy
10	Liver	1,2 X 1,2 X 0,3 cm	FFPE	Laparotomy

Supplementary Table 1. Information on metastases. The table is providing detailed information on the origin and dimensions of metastasis samples.

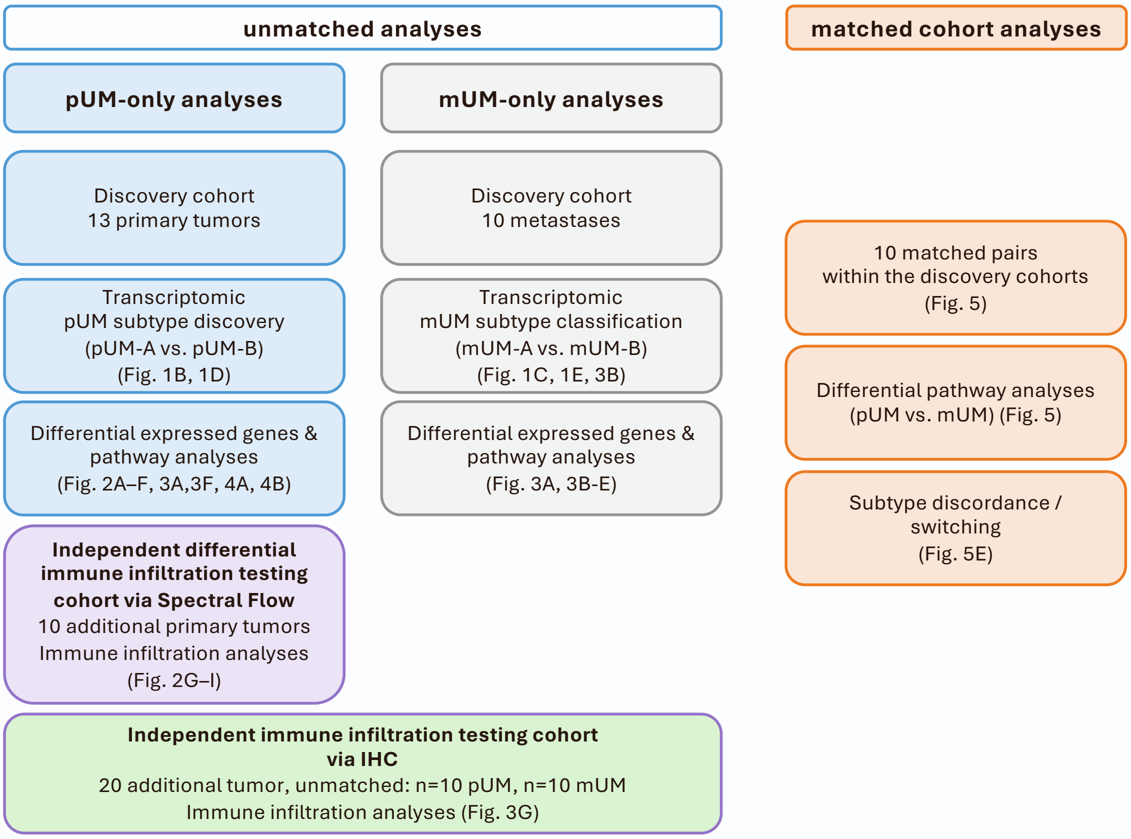

Figure S1. Sample Cohorts Overview. The workflow summarizes discovery and testing cohorts used for transcriptomic analyses, immune profiling by spectral flow cytometry and immunohistochemistry. It distinguishes unmatched discovery analyses from matched-pair analyses assessing subtype switching and pathway differences between primary tumors and metastases.
